# Networked Organizational Structure of Enterprise Information Security Management Based on Digital Transformation and Genetic Algorithm

**DOI:** 10.3389/fpubh.2022.921632

**Published:** 2022-06-23

**Authors:** Zhenpeng Di, Yun Liu, Shimei Li

**Affiliations:** School of Management Science and Engineering, Guangxi University of Finance and Economics, Nanning, China

**Keywords:** digital transformation, genetic algorithm, enterprise information security, multi-objective model, networked organizational structure

## Abstract

With the rapid development of society and economy, enterprises have also started digital transformation in order to follow the pace. Now-a-days, the development of enterprises is also faced with the risk of improper management of enterprise information security, so it is very necessary to study the networked organizational structure of enterprise information security management. The purpose of this paper is to study how to analyze the network organization structure of enterprise information security management based on genetic algorithm. This paper puts forward the difficult problems faced by enterprise information security, and how to prevent and solve the risks encountered in information security. In the experimental part of this paper, it can be seen that with the increase of the number of experiments, the work efficiency of the traditional network organization structure has increased from about 4.6 to about 17%. The work efficiency of the network organization structure based on genetic algorithm has risen from about 10 to about 50%. It shows that the work efficiency of the network organization structure based on genetic algorithm is much higher than that of the traditional network organization structure. It can be seen that the advantage percentage of enterprise information security management networked organizational structure with strong room for growth is 79–84%, and the percentage of cost savings is 75–82%. It can be seen that there are still many advantages of the networked organizational structure of enterprise information security management. The network organization structure based on genetic algorithm can not only improve the work efficiency of enterprises, but also improve the information security. Therefore, the network organization structure based on genetic algorithm is of great significance to the enterprise information security.

## Introduction

Although the establishment of enterprise information security management in China started relatively late, in recent years, the establishment of enterprise information security management has been highly appraised by governments at all levels, relevant departments and safety production departments. At this stage, China's enterprise information security management construction has achieved some important results, but it is still in the exploratory stage. A major benefit of digital transformation is that it provides data about customers, enabling businesses to improve the customer experience. From messaging customers with the right channel and message at the right time, to personalizing product websites, to digital transformation enabling businesses to give customers the experience they want.

In today's society, computers and networks are used more and more frequently, and people store a large amount of information on servers and hosts. For enterprises, computer network has gradually become the main information transmission carrier and channel. Network information security problems are also increasing, causing immeasurable losses and threats to corporate interests. Therefore, it is necessary to take necessary countermeasures and preventive measures for the security of data and information on the internal network.

The innovation of this paper is: (1) The theoretical knowledge of digital transformation and enterprise information security is introduced, and genetic algorithm is used to analyze how genetic algorithm plays a role in the research of enterprise information security management network organization structure. (2) The characteristics of enterprise information security management networked organizational structure and its advantages are investigated and analyzed, and finally it is concluded that the networked organizational structure of enterprise information security management based on genetic algorithm has more advantages.

## Related Work

With the development of enterprises, the network of information security management in enterprises has become more and more important. Bustamante F proposes a new method to solve the problem of information security management of industrial control system. His research shows the advantages of this approach to align recommendations for effective management of information security with strategic goals. The development of his research is divided into three different stages, all of which can well solve the problem of security information leakage. But he did not propose a specific solution, nor did he mention the specific content of the three stages (1). Zeng W has found that enterprise information security technologies have proven successful in protecting sensitive information in business organizations by providing effective access control mechanisms. However, this safety mechanism often reduces employee productivity as they spend time on tasks that are not related to the project. As a result, organizations must invest heavily in information security technology and then continue to incur additional costs. He starts by discussing a solution and using unproductive time to quantify the loss of worker productivity. Although he is aware of the harm brought by information security technology, there is no specific experimental object and experimental design to prove his conclusion (2). In order to construct an effective enterprise information security evaluation system, Liu S analyzed the factors affecting the enterprise information security level in the big data environment. He established a comprehensive evaluation model based on the main influencing factors affecting the level of enterprise information security in the big data environment. Although he raised the source of the problem and established the corresponding model, he did not elaborate the specific process of building the model, which made the credibility of the model very low (3). Shakirov MB found that automated systems play a key role in supporting the business processes of an enterprise. The widespread use of automated information systems for storing, processing and transmitting information has made the issue of their protection sensitive, especially given the global trend toward an increase in the number of information attacks leading to significant financial and material damage. He demonstrated the importance of auditing in the field of optoelectronic instrument information security. He discusses the stages and rules for conducting an information security audit, as well as the criteria for evaluating its results. He believes that information security auditing is one of the most effective tools for obtaining an objective assessment of information security threats. He not only raised the problem, but also solved the problem, but did not highlight the advantages of information security audit (4). Sizov A presents the results of a study aimed at determining the capabilities of future software engineers in securing enterprise information security activities to address the crises encountered. He developed a program to assess the level of professional competence formation of college students and conducted a comparative analysis of the acquired empirical data of two groups of elementary education students. Finally, it is concluded that in order to ensure enterprise information security, it is necessary to formulate special teaching conditions for the formation of future software engineer capabilities. The content and object of his experiment are very clear, but there is no experimental data, so it is difficult to support the conclusion of the experiment (5). Kaupadien L found that information security is one of the main concerns for businesses. To ensure proper management of information security, he has developed a series of information security management frameworks. The condensed information in the information security management framework is very important for small and medium enterprises, because such enterprises often lack the resources for information security expertise and in-depth analysis. Therefore, he uses the Analytic Hierarchy Process to construct the hierarchical quality of the information security management framework and its applicability in SMEs. Although he put forward specific solutions for the information problems of small and medium-sized enterprises, he neglected to propose methods for information management of large-scale enterprises (6). Kuzmicheva S found that in order to prevent possible dangerous events and meet the requirements of enterprise development, enterprises should establish security systems for key information structures to ensure their functions. He proposed a method for developing an information security analysis system based on machine learning. The method allows the analysis of a large number of events to make informed decisions about information security management, develops a list of the main sources of system and network information security incidents, and proposes a classification of incidents for further analysis using machine learning. But he did not put this method into practice, so its authenticity and reliability are not very strong (7). The goal of Il'Chenko LM's work is to identify and assess information security risks for typical distributed information systems in three controlled areas. He mainly emphasizes that the application of information security in the information system under consideration is to minimize the damage caused by security threats, aiming at ensuring the integrity and availability of the hardware and software complex of the information system. He established the basic requirements for information security risk assessment and treatment. But he did not describe in detail how to reduce the damage caused by security threats (8).

## The Concept of Enterprise Information Security and Genetic Algorithm

The popularization of computer networks brings convenience to people, but also brings many hidden dangers to people, especially information security (9–11). For enterprises, information security is the core of information management (12). The orderly development of the information structure is an important guarantee for the healthy development of an enterprise. The information structure is conducive to building a learning organization, and the organizational structure is the lever force to realize knowledge management. When the internal and external environment of the organization changes, the network organization can continuously adapt to the environment and continuously improve itself.

**Figure 1:**
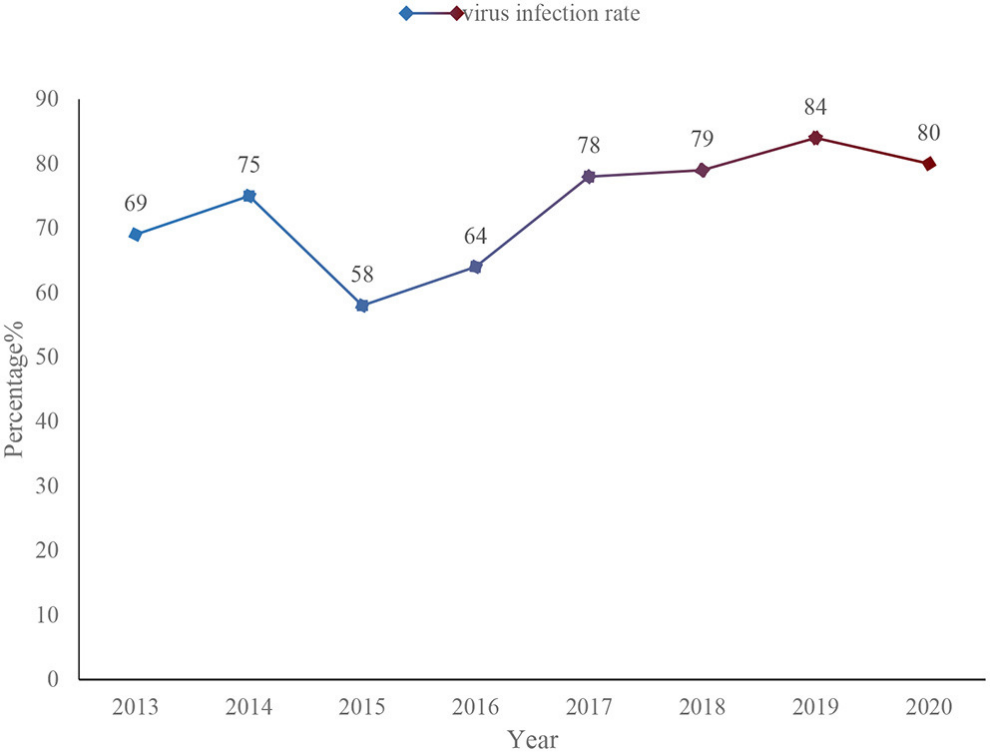
Virus incidence of information security incidents from 2013 to 2020.

This paper makes statistics on the virus incidence of information security incidents from 2013 to 2020, as shown in Figure 1.

As shown in Figure 1, from 2013 to 2020, the virus incidence rate of information security incidents has increased from 69 to 80%. Although there has been a drop in the middle, the fluctuation is not very large. The so-called safe information is the content that does not pose any threat to society and the network. It can prevent the transmission of dangerous information in the network and society, such as poisonous programming scripts, bad information, useless spam and so on. If this information is allowed to spread unrestrictedly, it will seriously threaten the stability and unity of the society, affect the normal operation of the system, and also occupy a lot of resources (13–15). The application of information security technology can effectively ensure the authenticity and integrity of information, and can also be well protected in terms of controllability and confidentiality.

**Figure 2:**
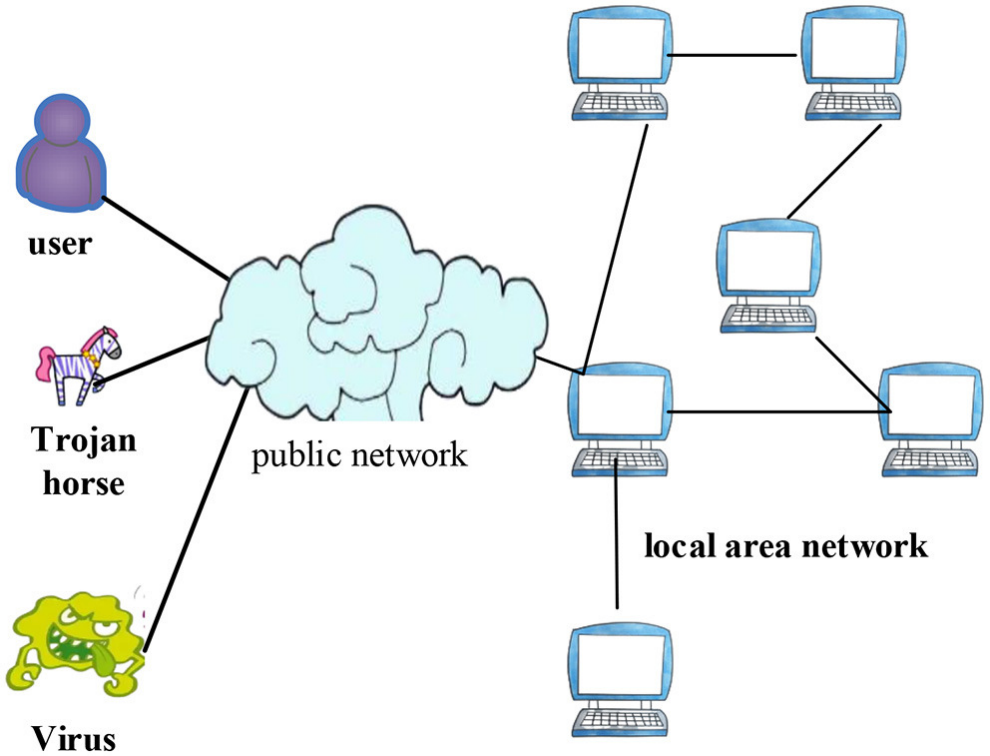
Traditional network information security management.

With the continuous improvement of computer system functions, the system configuration is also more and more complex, which may lead to huge losses. Such as stealing information resources and sensitive information in the system by various possible legal or illegal means. For example, monitoring the signal transmitted in the communication line, or intercepting useful information by using the electromagnetic leakage generated by the communication equipment during the working process. The traditional network information security management is shown in Figure 2.

**Figure 3:**
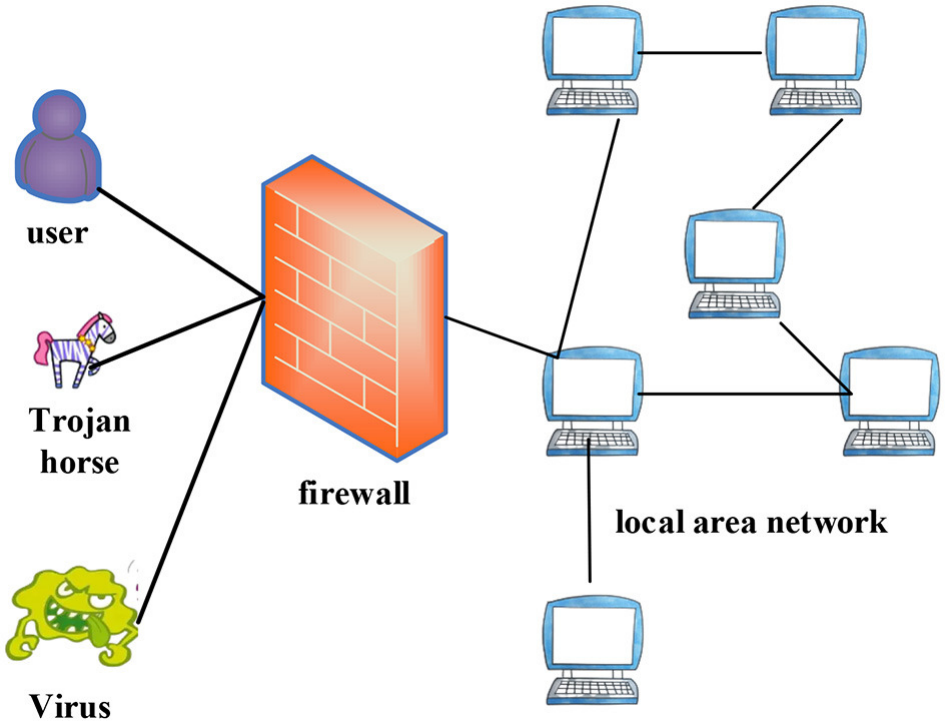
Reasonable enterprise information security design.

As shown in Figure 2, network security, especially in the practice of enterprise computer network information security management, has become the core issue of modern social development, and is also the main problem faced by enterprise managers and network information security managers. And reasonable enterprise information security organization design is an important means to achieve security management (16–18). A reasonable enterprise information security design is shown in Figure 3.

As shown in Figure 3, information security in today's society has become an important factor affecting enterprise security, which is directly related to the financial environment and ideology of enterprises, so information security has received more and more attention (19–21).

**Figure 4:**
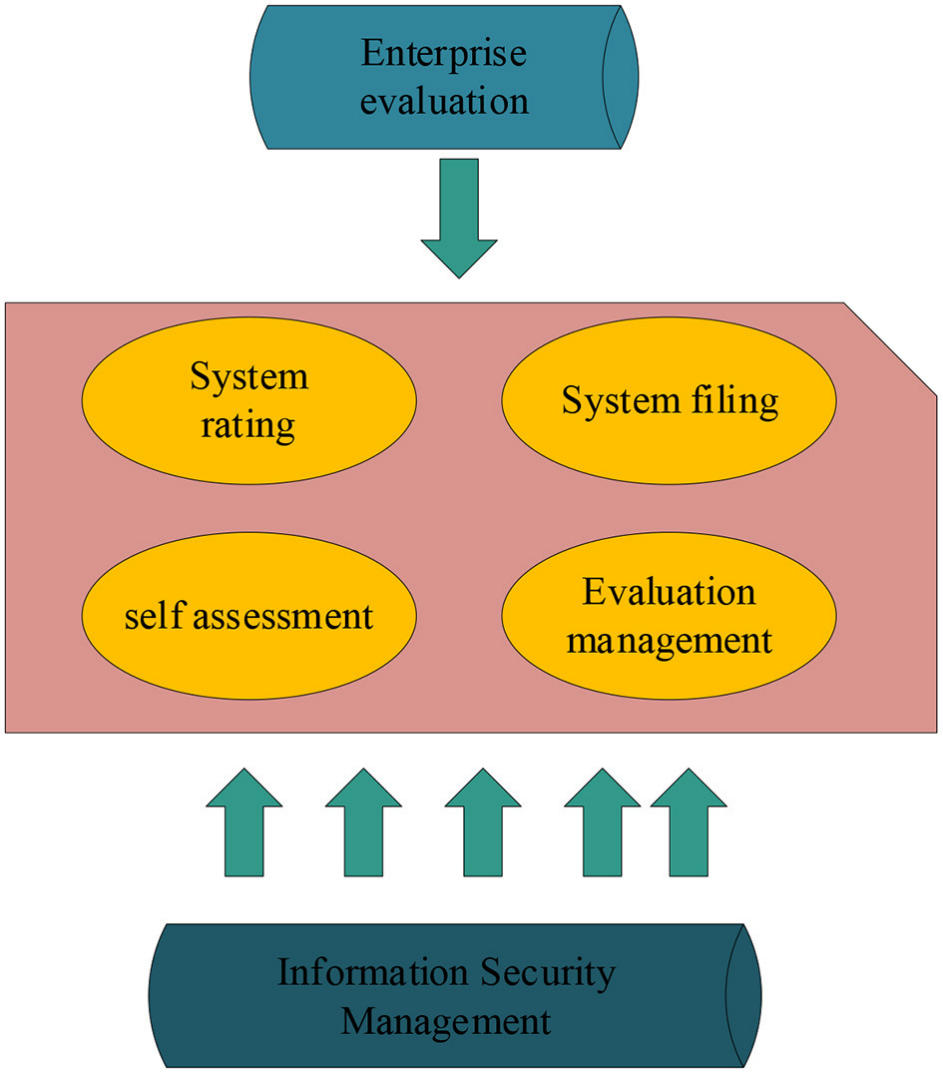
Functional module of compliance information security management.

The hierarchical protection compliance management function module supports the corporate headquarters and subordinate units to carry out important information security protection management work in accordance with the relevant policy standards for hierarchical protection, including related work activities such as data collection, rectification, and evaluation of each unit's information system (22–24). The functional module of compliance information security management is shown in Figure 4.

As shown in Figure 4, the platform has built-in level protection standard rules and related knowledge and experience, which provides effective support for the data reporting and analysis process. By summarizing the data, the basic situation related to the level protection of each unit can be statistically analyzed, and the security status can be compared by year and unit.

**Figure 5:**
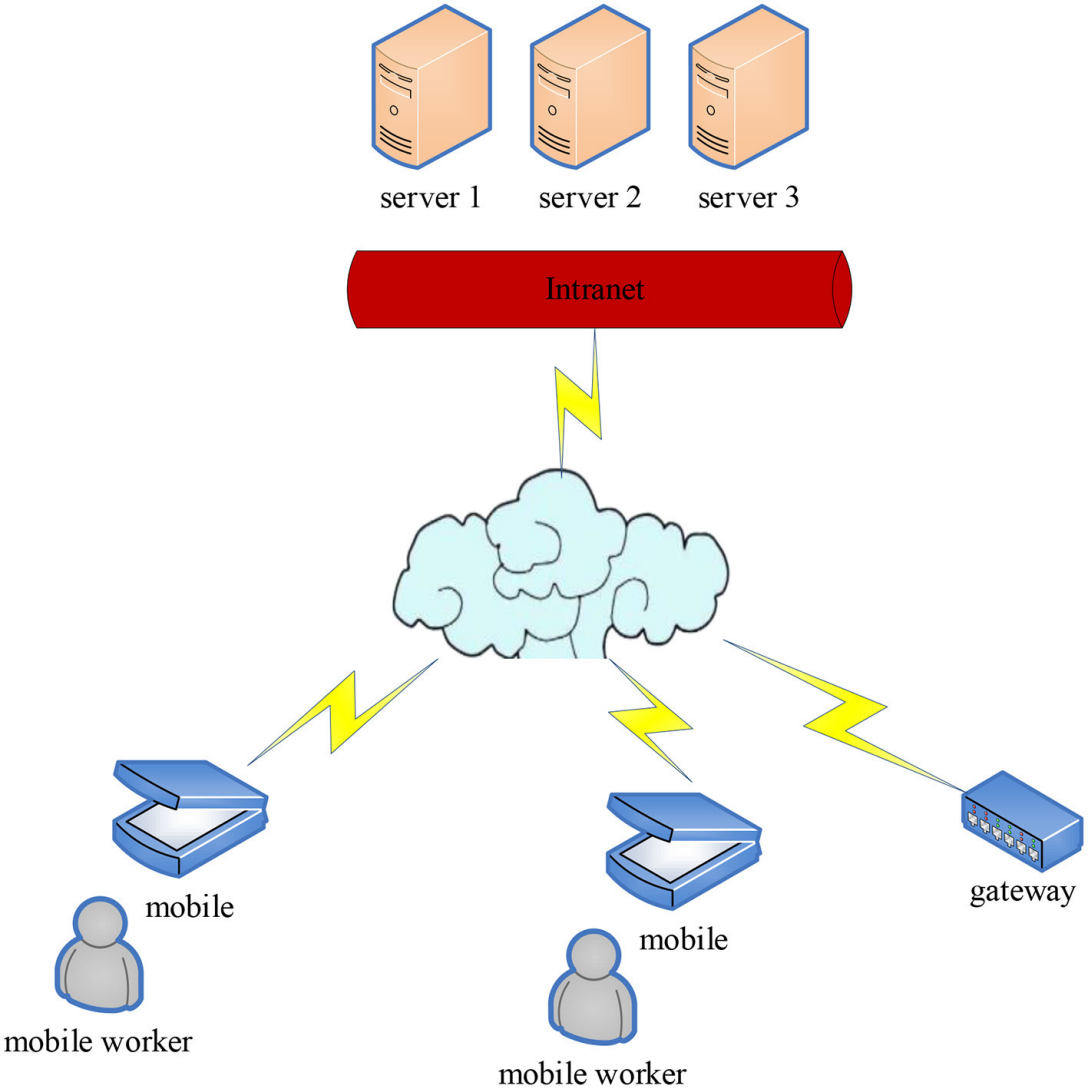
Corporate headquarters information management system.

The leaders of the information management department of the corporate headquarters can view and count all kinds of information related to the level protection work of each subordinate department in real time in the level protection compliance management module. It includes the basic information of the information system, the grading and filing of the information system, the self-assessment of the information system gap of each unit, and the assessment of the information system. The corporate headquarters information management system is shown in Figure 5.

As shown in Figure 5, due to the lack of talents and the limited understanding of informatization, the informatization of enterprises is faced with the problem of easy confusion. In order to solve these problems, enterprise personnel should not only improve security awareness, but also master the rules of enterprise informatization (25), establish an organizational structure that helps to promote informatization, and formulate scientific development plans, and implementation plans.

## Multi-Objective Model Based on Genetic Algorithm and Improved Genetic Algorithm

Genetic algorithm is designed and proposed according to the evolution law of organisms in nature. It is a computational model of the biological evolution process that simulates the natural selection and genetic mechanism of Darwin's theory of biological evolution. It is a method to search for the optimal solution by simulating the natural evolution process. As a new global optimization search algorithm, genetic algorithm is widely used in various fields because of its simple and practical characteristics, and has achieved good results, and has gradually become one of the important intelligent algorithms (26).

### Multi-Objective Model of Genetic Algorithm

Genetic algorithms are widely used in many fields to provide a general framework for solving complex system problems. This paper mainly introduces the application of genetic algorithm in enterprise information security management network (27). The role of genetic algorithm in intrusion detection is mainly to update the rule base. Through these rules, abnormal network connections can be found, and abnormal intrusions often exist in these abnormal connections.

(1) Objective function

In the multi-objective problems handled by genetic algorithm, all the objective problems are based on the form of *V*_min. *V*_min represents the minimization of the calculated vector, that is, each sub-objective in all vector objectives $f\;(a)={\left[f_{1}\;(a),f_{2}\;(a),...,f_{p}\;(a)\right]}^{T}$ belongs to the minimization problem (28), as shown in formula 1:


(1) $$\begin{array}{l} M{\text{inf}}_{1}\;\left(a\right)=\frac{-a_{1}}{a_{2}+a_{3}} \end{array}$$

The objective function is the target form that is pursued by the design variables, so the objective function is the function of the design variables, which is a scalar. In the engineering sense, the objective function is the performance criterion of the system. The sub-objective function *a*_1_ has both the function of maximization problem and the function of minimization problem, so it is necessary to transform the maximization problem in multi-objective. Just taking the negative number of the original problem and find the minimum value *M*inf_2_(*a*) (29), as shown in formula 2:


(2) $$\begin{array}{l} M{\text{inf}}_{2}\;\left(a\right)\;=\;-a_{1}\;-\;\frac{1}{a_{4}}\;-\;a_{5} \end{array}$$

Multi-objective problems are different from single-objective problems. In most cases, the sub-objectives *a*_1_, *a*_4_, and *a*_5_ in multi-objective problems are likely to conflict with each other. Therefore, in order to solve this kind of problem accurately, it is necessary to understand the definition of optimal solution.

**Figure 6:**
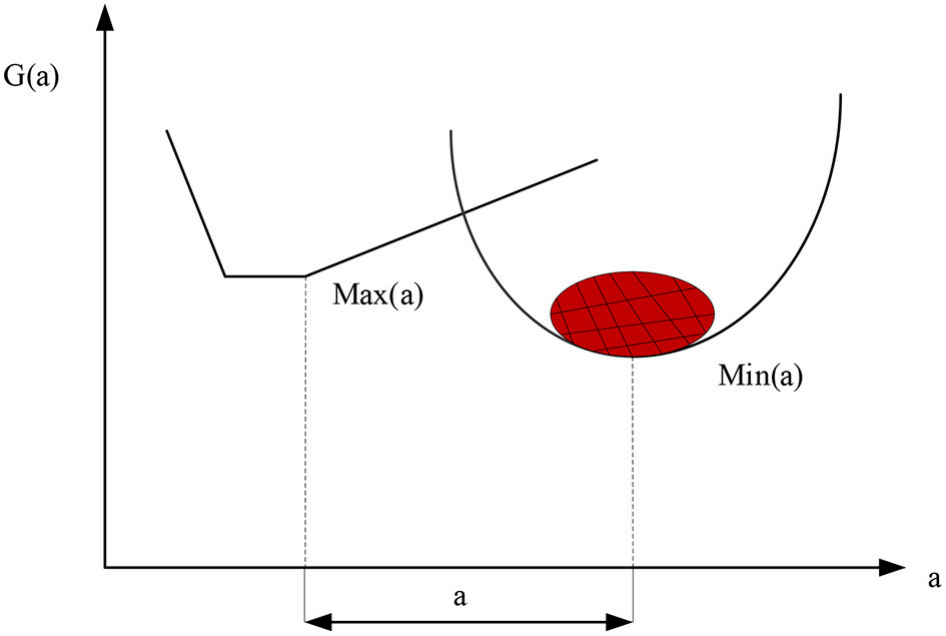
The optimal solution of the multi-objective model.

Usually the optimal solution is defined as the best solution that can be achieved technically without sacrificing any overall objective and each sub-objective. It represents an ideal solution that can be achieved by all overall and sub-goals. The optimal solution is simply an acceptable “not bad” solution in the set of model solutions for multi-objective problems (30). Meanwhile, the same multi-objective optimization problem may have multiple solutions, as shown in Figure 6.

As shown in Figure 6, when using the genetic algorithm to deal with multi-objective problems, how to deal with multiple objective functions is the primary problem to solve the model. Many scholars have proposed the method of weight coefficient, which assigns the corresponding weight to each sub-objective, and then combines the weighted calculation of each objective into a single objective function (31). The weight coefficient indicates the importance of an index item in the index item system. It expresses the influence of the change of this index item on the result when other index items remain unchanged. The size of the weight coefficient is related to the importance of the target.

In multi-objective optimization problems, the weight method is often used to obtain the optimal solution (32). The theoretical formulation of the method is as follows:

In a multi-objective problem model, if different weight coefficients *w*_*i*_ (*i* = 1, 2, ..., *P*) are assigned to each sub-objective *f*_*i*_(*a*), the single-objective function is shown in formula 3:


(3) $$\begin{array}{l} U\;\left(f\;\left(a\right)\right)\;=\;\overset{p}{\underset{i=1}{\sum }}w_{i}f_{i}\;\left(a\right) \end{array}$$

Among them, the size of *w*_*i*_ reflects the importance of the sub-objective function in the multi-objective function model, and whether the weight assignment is reasonable has a very important influence on the scientificity of the evaluation results.

**Figure 7:**
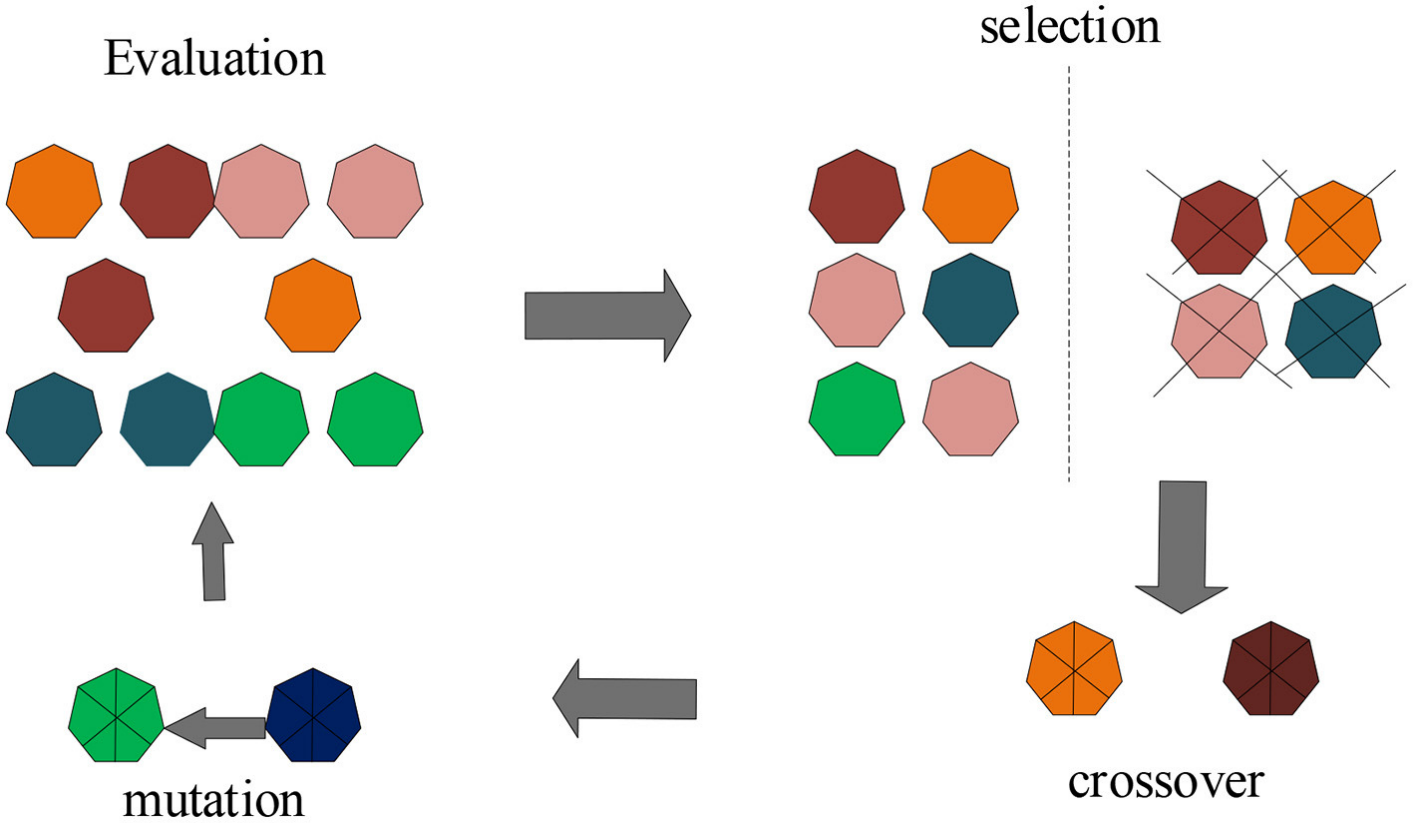
The operation process of the genetic algorithm.

The operation process of genetic algorithm is an iterative process. First determining the actual problem parameter set, and then follow the steps of initializing the population, coding, fitness calculation, and selecting the genetic strategy to iterate continuously until the required number of iterations is finally reached. The operation process of the genetic algorithm is shown in Figure 7.

As shown in Figure 7, the genetic algorithm is a cyclic iterative operation performed on the population. Before the operation, some initial groups need to be prepared as the initial search points of the cyclic operation, and these initial points are randomly generated by the computer. In the program coding, the *Crtbp* function of the genetic algorithm is called to create a binary initial population, and the format of the *Crtbp* function is as shown in formula 4:


(4) $$\begin{array}{l} Crtbp\;\left(Nind,NVAR*PRECI\right) \end{array}$$

Among them, *Nind* is the number of individuals, *NVAR* is the number of variables in the actual problem, and *PRECI* is the number of binary digits.

(2) Fitness function

The selection of the fitness function directly affects the convergence speed of the genetic algorithm and whether it can find the optimal solution. Because the genetic algorithm basically does not use external information in the evolutionary search, it is only based on the fitness function, and uses the fitness of each individual of the population to search. In genetic algorithm, fitness function is used to evaluate the survivability of individuals. Generally speaking, a good gene structure has a higher fitness value, which means it has a strong adaptability, and it is not easy to be eliminated in the operation.

In order to associate the fitness function with the pros and cons of an individual in the population, the fitness value in the genetic algorithm must be non-negative, and the direction of optimization is to increase the fitness value. The mapping from the objective function to the fitness function usually takes the following two different cases.

For the maximization problem, the mapping relationship with the objective function can be established as formula 5:


(5) $$\begin{array}{l} F\;\left(a\right)\;=\;\{\begin{array}{c} f\;\left(a\right)\;-\;C_{\min},f\;\left(a\right)\geq 0\\ 0,other \end{array} \end{array}$$

Among them, *C*_min_ can be either the input value or a theoretical minimum value, or it can be replaced by the minimum value of all current operation results.

For the minimization problem, the following method can be used for transformation, such as formula 6:


(6) $$\begin{array}{l} F\;\left(a\right)\;=\;\{\begin{array}{c} C_{\max}\;-\;f\;\left(a\right),f\;\left(a\right)\leq C_{\max}\\ 0,other \end{array} \end{array}$$

Among them, *C*_max_ can be either the input value, or a theoretical maximum value, or it can be replaced by the maximum value of all the current operation results, but at this time it will change continuously with the change of algebra.

(3) Selection operator

In view of the fact that the traditional selection operator cannot overcome the non-negative fitness problem when the genetic algorithm solves the optimal scheduling of the reservoir, a trigonometric function selection operator is proposed. The selection operation of the genetic algorithm simulates the natural law of “survival of the fittest” in the biological world, realizes individual selection, and improves the convergence performance and execution efficiency of the genetic algorithm.

The size of the population is denoted as *P* = {*x*_1_, *x*_2_, ..., *x*_*m*_}, where the probability of an individual *x*_*j*_ ∈ *p* being selected is formula 7:


(7) $$\begin{array}{l} P_{s}\;\left(x_{j}\right)\;=\;\frac{f\;\left(x_{j}\right)}{\overset{m}{\underset{i=1}{\sum }}f\;\left(x_{i}\right)} \end{array}$$

When the individual fitness value is larger, the probability of *f* (*x*_*j*_) being selected is also larger. In addition, the production expectation of an individual in the parent population is formula 8:


(8) $$\begin{array}{l} P_{s}\;\left(x_{j}\right)\;=\;\frac{f\;\left(x_{j}\right)}{\overset{m}{\underset{i=1}{\sum }}f\;\left(x_{i}\right)} \end{array}$$

**Figure 8:**
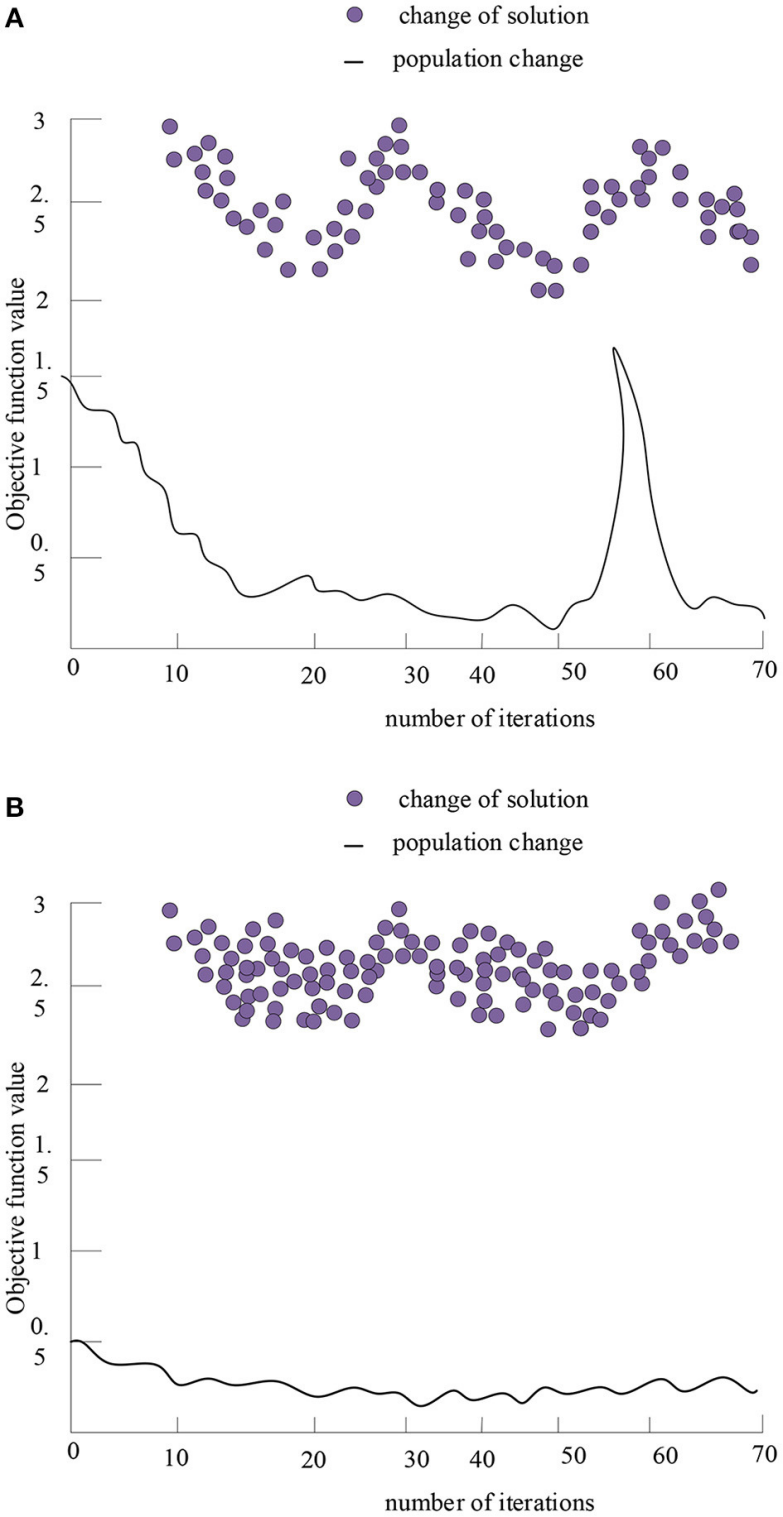
Optimal solutions and performance tracking of the second objective function and the third function after 70 iterations. **(A)** Optimal solution and performance tracking of the second objective function after 70 iterations. **(B)** Optimal solution and performance tracking of the third objective function after 70 iterations.

After using the fitness function and the selection operator to perform sub-iteration operations on the multi-objective function, the following operation results are obtained, as shown in Figure 8.

Figure 8 reflects the optimal solution of the second objective function and the third objective function. When only considering this objective function, the theoretical optimal solution is not very good. The third objective function is the simplest function among the three objective functions, the program quickly converges to the optimal solution and the fluctuation is small in the whole operation. It reflects that as long as the input of various resources is sufficient, the preparation for the next value creation process will be more adequate, and the more likely it will be to create more value.

In each iteration of the genetic algorithm, a new generation of hypotheses is generated based on the current population. The probability of choosing hypothesis Pr(*h*_*i*_) is as in formula 9:


(9) $$\begin{array}{l} \mathrm{Pr}\;\left(h_{i}\right)=\frac{Fitness\;\left(h_{i}\right)}{\sum_{j=1}^{p}Fitness\;\left(h_{j}\right)} \end{array}$$

Therefore, the probability Pr (*h*_*i*_) of a hypothesis being selected is proportional to its own fitness *Fitness* (*h*_*i*_).

### Improved Genetic Algorithm

Although the genetic algorithm can optimize the initial weight of the network, it also has the problem of premature convergence, that is to say, the genetic algorithm can play its global optimization role better in the initial stage of training. As the number of iterations increases, the optimization effect becomes less and less obvious.

Based on the above analysis, this paper proposes an improved genetic algorithm. Compared with the binary coding method commonly used in genetic algorithm, the improved genetic algorithm has the advantages of short individual length, simpler decoding method, and easy to solve the problem of different variable value ranges. Thus, the expression of chromosome diversity for the solution is increased, as in formula 10:


(10) $$\begin{array}{l} A=\{\begin{array}{c} x_{0},x_{1},x_{2},...,x_{m}\\ y_{0},y_{1},y_{2},...,y_{n}\\ c_{0},c_{1},c_{2},...,c_{p} \end{array} \end{array}$$

Among them, A is an individual in the solution space, x, y, and c are various types of subcode strings, m, n, and p are the dimensions of the subcode strings. The operations such as crossover and mutation can be carried out separately in the substring, thus enhancing the expression ability of the improved genetic algorithm for the problem.

According to the mathematical model established by genetic algorithm, the set of individuals is called the solution space. When generating the initial population, if the initial population can be uniformly distributed in the solution space, it is beneficial to the parallel search of the genetic algorithm. The initial population can be generated manually or randomly. This paper adopts the method of random algorithm generation. Introducing a symbolic function *D*(*a*) as in formula 11:


(11) $$\begin{array}{l} D\;\left(a\right)\;=\;\{\begin{array}{c} 0,a\neq 0\\ 1,a=0 \end{array} \end{array}$$

Assuming the penalty coefficient is f, the objective function 2 and the constraint condition 2 are combined into an objective function as formula 12:


(12) $$\begin{array}{l} f_{2}={\overset{n-1}{\underset{i\;=\;1}{\sum }}\;\left(\overset{m}{\underset{k\;=\;1}{\sum }}a_{ki}*a_{kj}\;-\;1\right)}^{2} \end{array}$$

In the process of solving the genetic algorithm, the multi-objective is first synthesized into a single-objective. There are many multi-objective synthesis methods, and the linear weighted summation method is used here, in which the weight is determined by the priority level of the target value and its own magnitude. Let the total target be f, and the weights are *w*_1_ and *w*_2_, respectively. Then the fitness function value of the individual is formula 13:


(13) $$\begin{array}{l} f\;=\;w_{1}f_{1}\;+\;w_{2}f_{2} \end{array}$$

This paper compares the efficiency of the genetic algorithm before and after the improvement under the condition that the optimization path is unchanged, as shown in Tables 1, 2.

**Table 1:** The efficiency of the genetic algorithm before the improvement when the optimization path is unchanged.

**Number of iterations**	**Search efficiency**	**Convergence speed**	**Optimized path**
5	46.98%	40.90%	29
10	43.72%	42.86%	27
15	50.74%	48.39%	25
20	53.04%	53.06%	24

**Table 2:** The efficiency of the improved genetic algorithm with the same optimization path.

**Number of iterations**	**Search efficiency**	**Convergence speed**	**Optimized path**
5	78.56%	69.05%	29
10	80.35%	74.68%	27
15	83.90%	79.06%	25
20	85.42%	82.94%	24

As shown in Tables 1, 2, when the optimization path is unchanged, the search efficiency of the genetic algorithm before the improvement is 46.98%, and the search efficiency of the improved genetic algorithm is 78.56%. For more complex problems, when the optimized path remains unchanged, although the improved genetic algorithm has high search efficiency, fast convergence speed, and can obtain better results, but there are still some problems. It shows that although the improved algorithm improves the performance of the genetic algorithm, there is still room for further improvement.

### Poisson Distribution Based on Genetic Algorithm

Poisson distribution is the probability distribution of the number of independent events occurring per unit time, and exponential distribution is the probability distribution of the time interval of independent events. In the information system, the used security measures can effectively reduce the risk of the information system, but the security measures cannot completely prevent the risk, which is defined here as formula 14:


(14) $$\begin{array}{l} S_{i}=1-\frac{U_{V}}{U} \end{array}$$

*U*_*V*_ represents the number of threats that actually cause losses to the system assets, U represents the total number of threats to the system, the probability of the threat is *Pt*, and the security risk *R*_*i*_ is formula 15:


(15) $$\begin{array}{l} R_{i}=U\times Pt_{i}\times Pv_{i} \end{array}$$

If the organizational structure adopts a security measure m, the security measure is applied to the organizational structure and becomes a part of the organizational structure, and a new threat factor j will be introduced. At this time, the risk *R*_*im*_ is formula 16.


(16) $$\begin{array}{l} R_{im}\;=\;U\times Pt_{i}\times Pv_{i}\times S_{i}\;+\;R_{j} \end{array}$$

Among them, *R*_*j*_ = *U* × *Pt*_*i*_ × *Pv*_*i*_ is the risk value introduced by security measures.

Poisson distribution: the possible values of the random variable x are 0,1,...,k,..., and are formula 17.


(17) $$\begin{array}{l} P\;\left(A\;=\;k\right)\;=\;\frac{e^{-\lambda }\lambda^{k}}{k!},k\;=\;1,2,...,n \end{array}$$

Where λ is a constant and satisfies λ ≥ 0, then the random variable x obeys the Poisson distribution with parameter λ, denoted as *A*^~^π (λ). The meaning of the parameter λ is the average occurrence rate of random events per unit time.

When the value of λ is large, the Poisson distribution can be converted into a normal distribution using the approximate formula as formula 18:


(18) $$\begin{array}{l} \lim\frac{k!}{\sqrt{2\pi k}k^{k}e^{-k}}\;=\;1 \end{array}$$

The times of occurrence of information security threats discussed in this paper all obey or approximately obey Poisson distribution. *N* (*t*) follows a Poisson distribution with parameter λ*t*, which is formula 19:


(19) $$\begin{array}{l} \mathrm{Pr}o\;\left(N\;\left(t\right)\;=\;k\right)=\frac{e^{\;-\;\lambda t}{\;\left(\lambda t\right)}^{k}}{k!} \end{array}$$

Finally, the quantitative model of information security risk assessment based on the occurrence of information system threats is defined as formula 20:


(20) $$\begin{array}{l} Rt\;=\;\overset{k}{\underset{k\;=\;1}{\sum }}\;\left(v\times \frac{e^{\;-\;\lambda }\lambda^{k}}{k!}\times pv\right) \end{array}$$

Poisson distribution is used to simulate the probability of threat occurrence. According to the characteristics of Poisson distribution, it can be known that the probability of threat occurrence increases with the increase of threat occurrence number when the number of events is less than that. When the number of threat occurrences is greater than λ, the probability of threat occurrence decreases with the increase of the number of threat occurrences.

## Experiment and Analysis of Networked Organizational Structure of Enterprise Information Security Management

### Characteristics, Advantages and Disadvantages of Networked Organizational Structure of Enterprise Information Security Management

This paper investigates the management strength of an enterprise in various departments, as shown in Table 3.

**Table 3:** A company's management efforts in various departments.

**Functional department**	**Information security work**	**Safety technology jobs**	**Audit**
Production section	33%	30%	32%
Sales section	28%	29%	26%
Finance section	15%	17%	19%
Human resources	14%	13%	15%
Information security department	10%	11%	8%

It can be seen from Table 3 that an enterprise supervises the production department, sales department, finance department, human resources department and information management department respectively. The enterprise invested the most in the production department, accounting for 33%, and invested the least energy in the information security department, accounting for 10%.

This paper investigates the characteristics of the network organization structure of 5 enterprises, and asks the enterprises to score by themselves, as shown in Table 4.

**Table 4:** The characteristic score of the network organization structure of 5 enterprises.

**Enterprise**	**Wholeness**	**Dynamism**	**Hierarchical**
A	7.6	6.7	8.5
B	8.4	6.9	8.2
C	8.2	6.4	7.9
D	8.1	7.2	8.1
E	7.9	7.4	8.0

As shown in Table 4, the characteristics of an enterprise's network organizational structure include integrity, dynamics and hierarchy. The five enterprises have relatively high scores for the three characteristics, the overall score is between 7.6 and 8.4, and the dynamic score is between 6.4 and 7.4.

(1) Integrity: Integrity means that the organizational process is a combination of at least two activities, because there are at least two activities in order to establish the mutual relationship between activities and to carry out circulation.

(2) Dynamic: The process is not static, but un-folds according to a certain relationship. The business process is to proceed to the next activity after the completion of one activity, but the organizational process can be based on the actual operation and organizational capability of the business process. The joint operation of multiple links, such as the monitoring of business processes, may exist in reorganization and optimization at the same time.

(3) Hierarchical: Hierarchy concretizes the concept of nesting, which means that some activities in the organizational process can also be regarded as sub-processes, which can decompose the activities of the organizational process.

**Figure 9:**
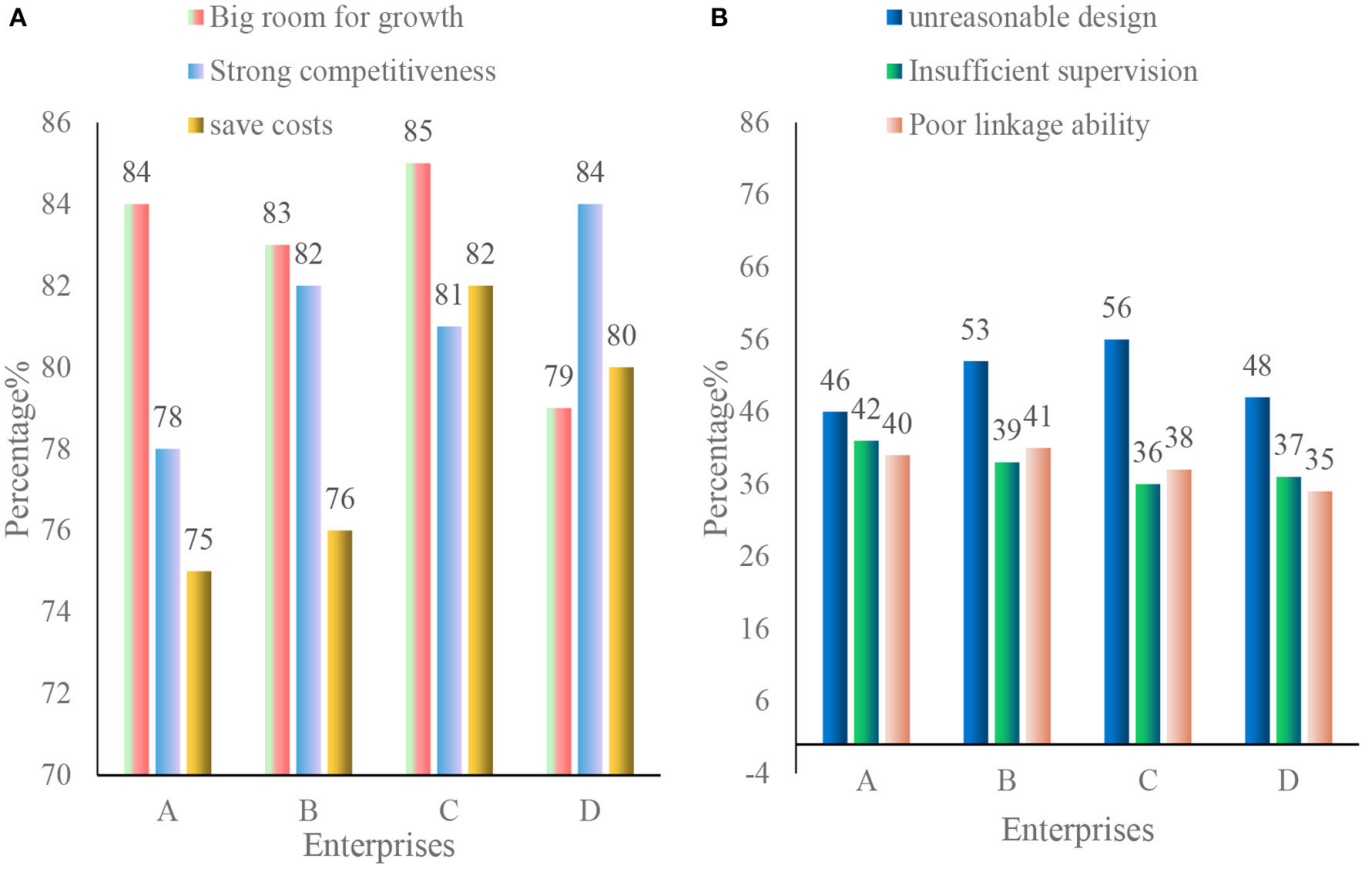
Advantages and disadvantages of a networked organizational structure for enterprise information security management. **(A)** Advantages of enterprise information security management networked organizational structure. **(B)** Disadvantages of the networked organizational structure of enterprise information security management.

This paper investigates the advantages and disadvantages of the networked organizational structure of enterprise information security management in four enterprises, as shown in Figure 9.

As shown in Figure 9, the advantages of the information network organization structure:

(1) Enterprises expand growth space through information innovation.

The formation process of enterprise information security network itself is a process of resource integration and cultural integration, forming a new market relationship between enterprises. Due to the formation of this new type of market relationship, the member enterprises of the information security network not only integrate the development of strategies, talents, learning ability and external resources on a comprehensive platform to form the competitiveness of enterprise value. The network not only forms the enterprise value network, but also expands the growth space for independent development.

(2) Saving costs and enhance competitiveness.

The network organization of market transactions between enterprises can reduce the adjustment costs within large enterprises and the production and organization costs of the entire value network. In addition, once the network organization is formed, management adjustments can be replaced with modular integration within the enterprise, thereby saving organizational costs.

(3) Network externalities promote the development of enterprise agglomeration.

The members of the enterprise information security network can share favorable resources such as information and technology by jointly complying with the interface standard of the value module, showing a strong network externality. The integration of enterprise organizations in the context of the network helps the interaction and coordination of functional elements among enterprises, promotes the development and production of technical products, and realizes the best final products.

The disadvantages of enterprise information management organizational structure are as follows:

(1) The design of the internal information security organization of the enterprise is unreasonable.

For enterprises, information security is multifaceted and complex. In order to ensure the formulation and implementation of information security management plans, it is necessary to establish an appropriate information security management organization. As a result, security risks still exist and security plans cannot be implemented. Security breaches can occur if the owners of the information and their responsibilities are not clearly defined.

(2) The enforcement and supervision of the enterprise information security organization is insufficient.

Most companies have established information security strategies, but due to unclear job responsibilities for information security and other reasons, information security managers cannot use technical or non-technical means to monitor and coordinate when abnormal situations occur, which leads to problems.

(3) Poor linkage ability of enterprise information security organization.

In the case of security problems, the cooperation mechanism of social members related to maintaining enterprise information security is not smooth, the cooperation ability is low, and the countermeasures to prevent the infringement of enterprise information security are insufficient.

### Measures to Promote the Development of Enterprise Information Security Organizations

**Figure 10:**
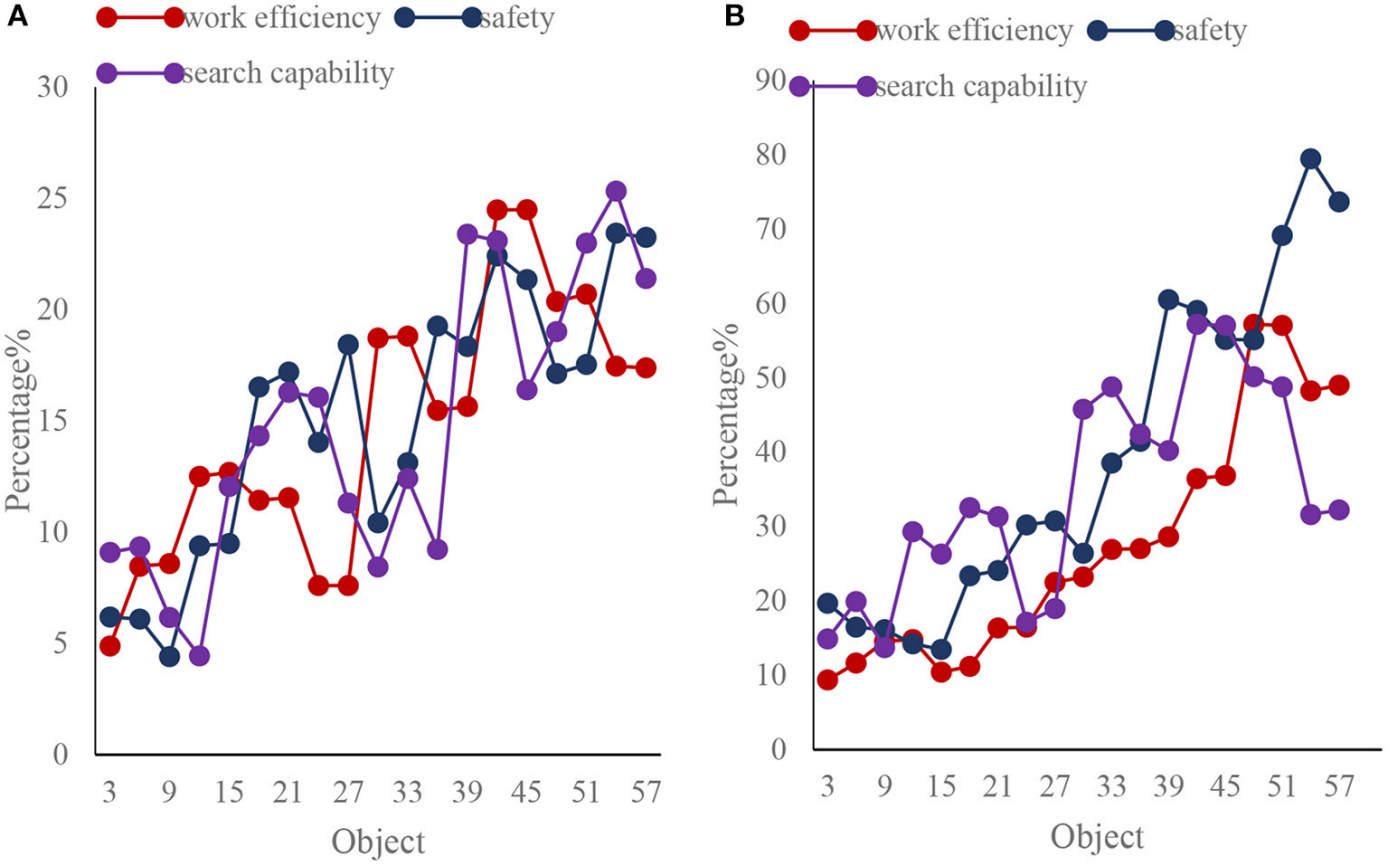
Comparison of two types of enterprise information security management networked organizational structures. **(A)** Traditional network organization structure. **(B)** Network organization structure based on genetic algorithm.

This paper investigates and compares the work efficiency, security and searchability of the traditional network organization structure and the network organization structure based on genetic algorithm, as shown in Figure 10.

As shown in Figure 10, the work efficiency, security and searchability of the traditional network organization structure are also slowly rising, but the work efficiency, security and searchability of the network organization structure based on genetic algorithm not only increase rapidly, but also greatly. It shows that the work efficiency, security and searchability of te network organization structure based on genetic algorithm have more advantages than the traditional network organization structure work efficiency, security, and searchability.

The countermeasures of the organization to promote information security management are as follows:

(1) Establishing an alliance-based information security management organization based on cooperation.

A cooperative information security management organization is a typical virtual organization. In this virtual organization, the nature of cooperation between enterprises and other members is diverse, and enterprises need to design various cooperation methods to cooperate.

(2) Countermeasures for establishing enterprise information security organization.

Establishing an information security leading group: the information security leading group is the highest leadership decision-making body for information security work within the organization. It does not cooperate with any department and is directly responsible for the top leadership of the organization. This matrix structure of the information security leadership group helps reduce bureaucracy.

(3) Strengthening the awareness of safety precautions and improve the quality of personnel.

First of all, we must pay attention to network security ideologically. No matter what position we are in, all employees of the enterprise must deeply understand the importance of computer information network.

## Discussion

This paper analyzes how to research the network organization structure of enterprise information security management based on genetic algorithm. This paper expounds the related concepts of digital transformation and genetic algorithm, focuses on the related theory of enterprise information security, and explores the method of network organization structure design of enterprise information security management, and through experiments to discuss the influence of enterprise information security management network organization structure on enterprise development.

This paper also uses a multi-objective model based on genetic algorithm. With the increasing application scope and importance of genetic algorithm, many scholars have begun to match genetic algorithm with real application scenarios and put forward feasible algorithms. The research and analysis of the genetic algorithm is actually a foundation for the research on the network organization structure of enterprise information security management in the experiment part of this paper.

Through experimental analysis, this paper shows that with the rapid development of enterprises, enterprises should find ways to enhance their competitiveness. This requires enterprises to network information security management through digital transformation, reduce workload, and save costs. Finally, it can be seen that the networked organizational structure of enterprise information security management can not only save costs, but also improve the competitiveness and cohesion of enterprises.

## Conclusions

In order to solve the problem of enterprise information security management encountered in the digital transformation of modern enterprises, this paper proposes a research on the network organization structure of enterprise information security management based on genetic algorithm. This paper expounds the basic concepts of genetic algorithm and enterprise information security, and introduces the importance of enterprise information security in detail. In the method part, this paper proposes a genetic algorithm. Based on the genetic algorithm, the objective function, fitness function, etc. are proposed. Aiming at the shortcomings of the traditional genetic algorithm, an improved genetic algorithm is proposed. After a simple experimental comparison, it is found that the improved genetic algorithm has stronger search ability and faster convergence speed. In the experimental part, this paper analyzes the characteristics, advantages and disadvantages of the organizational structure of enterprise information network, and proposes corresponding solutions according to the shortcomings of the organizational structure of enterprise information network. Under the background of faster and faster economic development, the research on the network organization structure of enterprise information security management is of great significance to the development of enterprises.

## Data Availability Statement

The original contributions presented in the study are included in the article/supplementary material, further inquiries can be directed to the corresponding author.

## Author Contributions

ZD: writing—original draft preparation. YL and SL: editing data curation. Both authors contributed to the article and approved the submitted version.

## Funding

This work was supported by ZD Science Research Startup King (K9-9999-15-00-00-015), Funding for the Construction of Management Science and Engineering Discipline of Guangxi Institute of Finance and Economics, Guangxi Higher Education Undergraduate Teaching Reform Project 2020 (2020JGZ147), and Planning Fund Project of Ministry of Education (20YJA790039).

## Conflict of Interest

The authors declare that the research was conducted in the absence of any commercial or financial relationships that could be construed as a potential conflict of interest.

## Publisher's Note

All claims expressed in this article are solely those of the authors and do not necessarily represent those of their affiliated organizations, or those of the publisher, the editors and the reviewers. Any product that may be evaluated in this article, or claim that may be made by its manufacturer, is not guaranteed or endorsed by the publisher.
